# Mycobacterium vaccae as Adjuvant Therapy to Anti-Tuberculosis Chemotherapy in Never-Treated Tuberculosis Patients: A Meta-Analysis

**DOI:** 10.1371/journal.pone.0023826

**Published:** 2011-09-06

**Authors:** Xiao-Yan Yang, Qun-Fei Chen, You-Ping Li, Si-Miao Wu

**Affiliations:** 1 Chinese Evidence-Based Medicine Center/The Chinese Cochrane Center, West China Hospital, Sichuan University, Chengdu, China; 2 West China Medical School, Sichuan University, Chengdu, China; Fundació Institut Germans Trias i Pujol, Universitat Autònoma de Barcelona CibeRES, Spain

## Abstract

**Objective:**

To evaluate the effectiveness and safety of heat-killed *M.vaccae* added to chemotherapy of never-treated tuberculosis (TB) patients.

**Methods:**

The databases of Medline, Embase, Biosis, Cochrane Central Register of Controlled Trials, SCI, CBM, VIP and CNKI were searched. Randomized controlled trials (RCT) and Controlled clinical trials (CCT) comparing *M.vaccae* with or without a placebo-control injection as adjuvant therapy in the chemotherapy of never-treated TB patients were included. Two reviewers independently performed data extraction and quality assessment. Data were analyzed using RevMan 5.0 software by The Cochrane Collaboration.

**Results:**

Fifty four studies were included. At the end of the follow-up period, Pooled RR (Risk Ratio) and its 95% CI of sputum smear conversion rate were 1.07 (1.04, 1.10) in TB patients without complications, 1.17 (0.92, 1.49) in TB patients with diabetes mellitus, 1.02 (0.94, 1.10) in TB patients with hepatitis B, and 1.46 (0.21, 10.06) in TB patients with pneumosilicosis. In elderly TB patients the RR was 1.22 (1.13, 1.32). Analysis of each time point during the follow-up period showed that *M.vaccae* could help to improve the removal of acid-fast bacilli from the sputum, and promote improvement of radiological focal lesions and cavity closure. Compared with the control group, the differences in levels of immunological indicators of Th1 such as IL-2 and TNF-α were not statistical significant (P = 0.65 and 0.31 respectively), and neither was that of IL-6 produced by Th2 (P = 0.52). An effect of *M.vaccae* of prevention of liver damage was found in TB patients with hepatitis B (RR 0.20 and 95% CI (0.12, 0.33). No systemic adverse events were reported.

**Conclusion:**

Added to chemotherapy, *M.vaccae* is helpful in the treatment of never-treated TB patients in terms of improving both sputum conversion and X-ray appearances.

## Introduction

Inactivated *M.vaccae* (MV), a heat-killed vaccine derived from a non-tuberculosis mycobacterium, is the only immunotherapeutic agent recommended by WHO in the Tuberculosis Strategic Development Plan of 1991 [1]. Tuberculosis (TB) is characterized by the combined activity of T-helper type 2 (Th2) lymphocytes with T-helper type 1 (Th1) lymphocytes [2]. *M.vaccae* can enhance cellular immune function through induction of Tr (regulatory T lymphocytes) [3] and by promoting macrophage effects. It was found in an earlier study that treatment of TB patients with *M.vaccae* can enhance Th1 and switch off the Th2 response [4]. As Th1 lymphocytes can provide protective immunity [2], it is crucial that immunotherapy for TB should swing the Th1/Th2 balance. Systematic reviews, which aimed to investigate *M.vaccae* immunotherapy as an adjunct to anti-TB treatment in previously treated patients [5] and multidrug-resistant tuberculosis (MDR-TB) patients [6], and as a preventive agent for people at high risk [7] have already been published and show that *M.vaccae* is safe and well tolerated, and can shorten the treatment course and promote conversion of clinical indicators. However, no systematic review is available for its use in never-treated TB patients, who are an important group of people for TB control and prevention in the community. Diagnosis and treatment of these people are essential to TB control as they are a source of infection until they die. From January to September 2009, about 750,000 TB patients were discovered and treated in China, among whom never-treated patients accounted for almost a half (340,000) [8]. In this paper, we explore the effects of *M.vaccae* as adjuvant therapy for never-treated TB patients.

There are two available preparations of *M.vaccae*, the original one developed in the U.K. is prepared from the rough variant of a selected strain, grown on a non-antigenic medium until the stationary phase. The washed organisms are suspended in borate-buffered saline, diluted to 10 mg (wet-weight)/ml, autoclaved and stored in vials for use. This reagent is either administered by intradermal injection or by mouth as capsules. The dose by either route is 1 mg of killed bacilli. The second, prepared in China, is made from the type strain of *M.vaccae*. It is 22.5 µg powder stored in vials for use, administered by intramuscular injection.

## Methods

### 1. Study eligibility criteria (PICOS)

#### 1.1 Participants (P)

Never-treated TB patients with sputum smear positive for acid-fast bacilli or culture positive for Mycobacterium tuberculosis (M. tuberculosis).

#### 1.2 Intervention (I)

Intervention: Inoculation with M.vaccae as an adjunct to standard anti-TB treatment.

#### 1.3 Control (Comparison) (C)

Control: Injection with a placebo plus anti-TB treatment, or anti-TB treatment alone.

#### 1.4 Outcome (O)

Primary outcomes: Conversion to sputum smear or culture negativity. Secondary outcomes:

- Change in clearance of X-rays: complete absorption; marked absorption (focal change>1/2); absorption (focal change<1/2); unchanged; or deterioration.
- Cavity closure rates: closure; unchanged;enlargement.
- Immunological indicators: changes in levels of markers of Th1(Tumor necrosis factor alpha [TNF-α], interferon-gamma [IFN-γ], interleukin-2 [IL-2]) and Th2 (interleukin-4 [IL-4], interleukin-6[IL-6]).
- Adverse events: such as local induration at the injection site, sore arm, skin breakdown, fever.

#### 1.5 Study design (S)

The studies selected for analysis were either Randomized controlled trails (RCT) or controlled clinical trials (CCT) without randomization. We did not use these terms as a restriction when searching the database, but filtered the articles by reading the abstract (and when necessary, the full length article, or by contacting the authors) in order to classify the studies.

### 2. Search strategy

English databases of the Cochrane Controlled Trials Register, MEDLINE, EMBASE, BIOSIS, SCI and Chinese databases of CBM, CNKI, VIP were searched till December 2010 using keywords of *Mycobacterium vaccae* and tuberculosis, without limitation of language, and the references of eligible studies were also searched. When the full length article were not available form the databases, we contacted the author asking for it.

### 3. Quality assessment in individual studies

Two reviewers (Q-fei Chen, S-miao Wu) independently performed data extraction and quality assessment. Four items were used to assess the quality of included studies based on Cochrane Collaboration recommended criteria: Adequate sequence generation, Allocation concealment, Blinding, and addressing the problem of incomplete outcome data.

### 4. Risk of bias across studies

The selective reporting within studies was assessed by answering whether the results were fully reported as the study was pre-specified (for example, if all the results were reported at all follow-up time points).

### 5. Statistical analysis

Statistical analysis was carried out using Revman 5.0 software. All studies were grouped by TB patients with different complications without consideration of variations in the anti-TB treatment regimens. Subgroups were delineated according to different time points during follow-up period. The heterogeneity test for the included studies was applied and p values of less than 0.05 were considered as statistical significance. The fixed effect model was applied in subgroups without heterogeneity, and in the others the random effect model was applied. G±1.96S_G_ (G-Geometric mean, S_G_ -standard error of geometric mean) was used to describe continuous variables and Risk Ratios (RR) and their 95% confidence interval (95%CI) were used for binary variable. Results were described for data that could not be combined and for safety evaluation.

## Results

### 1. Description of studies and quality assessment

**Figure 1:**
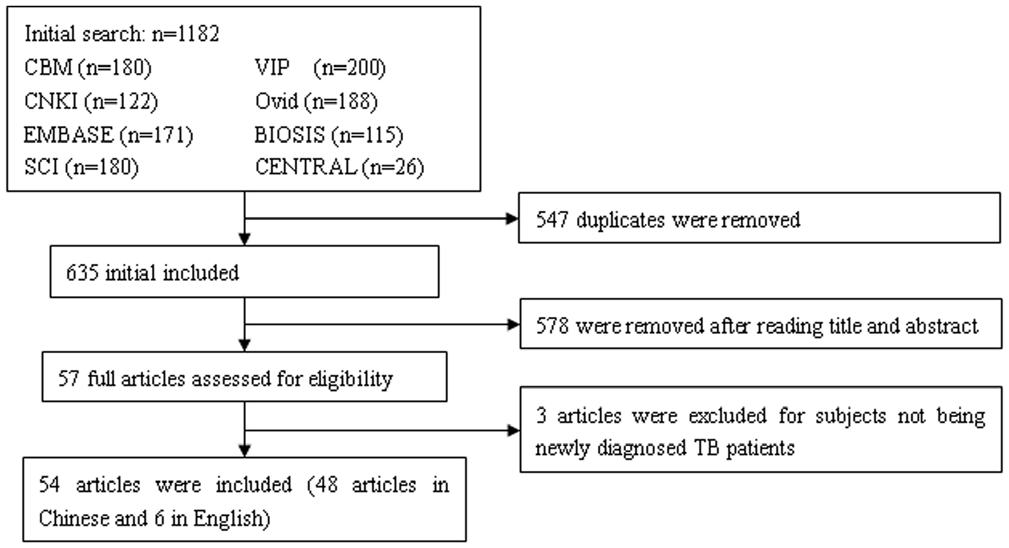
Flow diagram of study searching and selection process.

The initial search extracted 1182 articles, and after selection, 54 met the inclusion criteria among which 48 were in Chinese and 6 in English (Figure 1).

Table S1 shows the characteristics of the included studies.

Forty eight trials [9]–[56] were conducted in China and six [57]–[62] were carried out in Romania, South Africa, South American (Argentina), Uganda, and Zambia. Trials carried out in China included subjects with diabetes mellitus [39]–[45], hepatitis B [46]–[48] and pneumoconiosis [49], [50]. Studies in other countries included TB patients who were infected with the Human Immunodeficiency Virus (HIV) [58], [61].

The ages of the TB patients (excluding studies specifically on older patients [51]–[56]) ranged from 18 to 65. The TB patients in the studies of older patients [51]–[56] were all above 65 years.

In trials carried out in China, patients were inoculated with multiple doses of *M.vaccae*. Immunotherapy was generally administered as a dose of 22.5 µg per 1or 2 weeks and the duration of administration and the interval between doses varied. In trials outside China, a single dose of 0.1 ml of heat-killed *M.vaccae* containing 10^9^ organisms [57]–[61] was used, and in still others, three doses [62] were applied. The time of initiation of administration of *M.vaccae* in relation to chemotherapy varied between studies: 19 articles reported that *M.vaccae* was first inoculated at the end of the 2^nd^ week, 7 articles at the end of the 1^st^ week, and 7 articles on the 1^st^ day.

According to the Guidelines for the Implementation of Tuberculosis Control in China, the standard anti-TB treatment regimen used in China was 2 months of daily Isoniazid, Rifampicin, Pyrazinamide, with or without Streptomycin, followed by 4 months of Isoniazid and Rifampicin (2HRZE(S)/4HR) , see Table S1.

Table S2 shows the quality assessment of the included studies. The quality of the trials conducted abroad was higher than those carried out in China. After contacting the author, only 2 articles [32], [49] carried out in China got “yes” in “Adequate sequence generation” and no articles in China got “yes” in Allocation concealment. Among 6 studies carried out abroad, 5 [57], [58], [60]–[62] got “yes” in “Adequate sequence generation”, 5 [57], [58], [60]–[62] got “yes” in “blinding” and 3 [58], [60], [61] got “yes” in “Allocation concealment”.

The results at all follow-up time points which was pre-specified were fully reported in included studies. But the time points for the results of interest varied across studies, see Table S3, Table S4, and Table S5.

### 2. Primary outcomes of the effects of intervention

#### 2.1 Never-treated TB patients without complications

**Figure 2:**
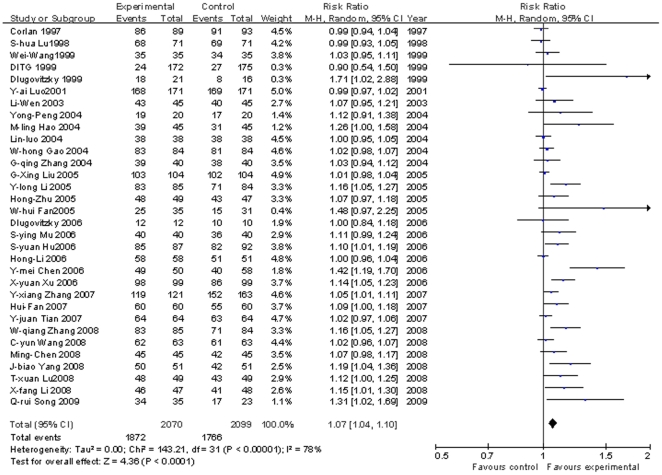
Meta analysis of sputum smear negative conversion rates in TB patients. Vertical line indicates no difference between the compared two groups (MV vs. control). Squares indicate point estimates of risk ratio (RR) in each individual study, the size of the squares indicates the weight of the corresponding study in the meta-analysis, 95%CIs of point estimates are shown by horizontal lines. Pooled RR and its 95%CI are shown by diamond shape. (It is the same in Figures 3, 4, 5, 6.)

Sputum smear conversion rate: Figure 2 shows the conversion rates of sputum smears to acid-fast bacilli negative (AFB−) of the *M.vaccae* recipient groups compared with the control groups receiving chemotherapy, with or without a placebo injection. The random effect model was applied at the end of the follow-up period (1–6 month after the first MV injection), the pooled RR and its 95% CI was 1.07 (1.04, 1.10), with statistical significance (P<0.0001). The pooled RR and its 95% CI of each follow-up month (1 to 6 months) are shown in Table S3. Although the anti-TB treatment regimens were different, the overall effects were all statistically significant through one to six months of follow-up (P≤0.05).

**Figure 3:**
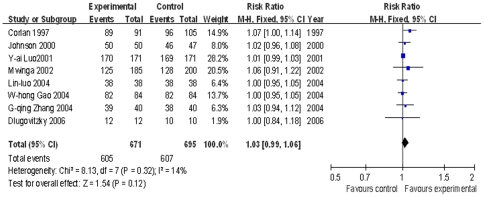
Meta analysis of sputum culture negative conversion rate in TB patients at the end of following up period.

Sputum culture negative conversion rate: In eight trials [12], [14], [16], [18], [57], [60]–[62] the sputum culture negative conversion rate was determined. The pooled RR and 95% CI at the end of the follow-up period were 1.03 (0.99, 1.06), without statistical significance (P = 0.12) (Figure 3). Further analysis of each follow-up time point at 1,2 4 and 6 months after injection showed that meta analysis at 1 and 2 months were statistically significant (P<0.05) but not significant at 4 and 6 months (P = 0.87 and 0.12 respectively) , see Table S3.

#### 2.2 Sputum smear conversion rates of TB patients with complicating medical conditions

**Figure 4:**
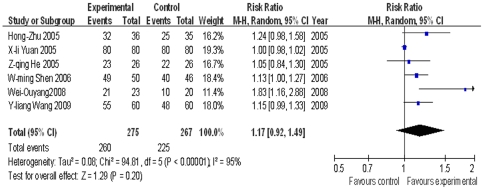
Meta analysis of sputum smear negative conversion rates in TB patients with diabetes at the end of follow-up period.

TB patients with diabetes mellitus: Six trials [39], [41]–[45] were based on TB patients with diabetes mellitus, with follow-ups at 1, 2, 3 and 6 months. The random effect model was applied at the completion of follow-ups. The pooled RR and 95% CIs were 1.17 (0.92, 1.49), without statistical significance (P = 0.20) (Figure 4). Meta analysis of sputum smear AFB− rate for each follow-up period is shown in Table S3. The overall effects of 2, 3 and 6 months were statistically significant (P = 0.009, 0.0001 and 0.01 respectively), while, it was not statistically significant at 1 month (P = 0.08).

TB patients with hepatitis B: In three trials [46]–[48] in which the smear AFB− rate at 6 months was determined, the pooled RR and 95% CI were 1.02(0.94,1.10), the P value of the overall effect was 0.65 without statistical significance , see Table S3.

TB patients with pneumosilicosis: Two trials [49], [50] reported the smear AFB− rate at 3 months and meta-analysis showed that pooled RR and 95%CIs were 1.46(0.21,10.06), without statistical significance (P = 0.70) , see Table S3.

#### 2.3 Sputum smear negative conversion rates of elderly TB patients

**Figure 5:**
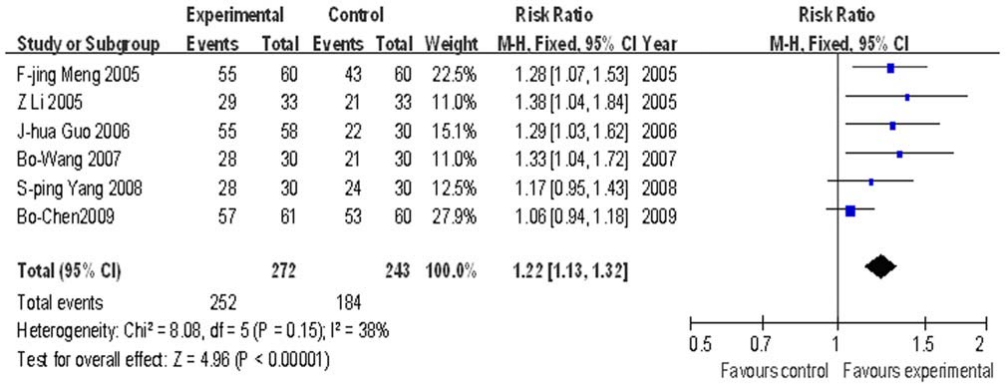
Meta analysis of sputum smear negative conversion rates in elderly TB patients at the end of follow-up period.

Six trials [51]–[56] reported the immunotherapyeutic effects of M.vaccae on elderly TB patients. Their combined result at completion of the follow-up period is shown in Figure 5. The fixed effect model was applied and the pooled RR and 95% CI were 1.22 (1.13, 1.32). The test for overall effect was statistically significant (P<0.00001). The subgroup results for each follow-up time point are shown in Table S3, where it can be seen that the overall effect at each follow-up time point was statistically significant (P<0.05) (except at 3 months, P = 0.61).

### 3. Secondary outcomes

#### 3.1 Meta analysis of radiological focal lesion changes and cavity closure rates

Table S4 shows that both complete absorption and marked absorption were significantly improved in M.vaccae recipients at 2, 4 and 6 month follow ups in never-treated TB patients without complications (P<0.05).

In TB patients with diabetes mellitus, marked absorption and absorption of focal lesions were statistically significant at the 6 month follow up, and in TB patient with HBsAg^+^, marked absorption of focal lesions was statistically significant at 6 months. In studies of elderly TB patients, marked absorption and absorption of lesions were statistically significant at 2, 4 and 6 month follow-ups.

Table S5 shows that cavity closure rates were significantly different between treatment groups at 2, 4 and 6 month follow-up periods in never-treated TB patient without complications (P<0.05). In TB patients with diabetes mellitus or HBsAg^+^, closure of cavities was significant at the 6 month follow-up. In TB patient with pneumosilicosis, the cavity closure rate was significant at the 6 month follow-up. In elderly TB patients, the difference in cavity closure between the two treatment groups was significant at the 2 and 4 months follow-up, but not significant at the 6 month follow-up.

#### 3.2 Immunological features

Immunological factors induced by Th1 lymphocytes: Serum (or plasma) IL-2: values were reported in 2 studies [10], [14]. It was found that IL-2 increased in the immunotherapy group compared with control group, but did not reach statistical significance in either study (P>0.05, data not shown). Also there was no statistical difference between the immunotherapy and placebo groups of the 2 studies, when they were combined (P = 0.65), see Table S6.

IFN-γ: In two trials [58], [60] it was shown that IFN-γ levels were elevated in the immunotherapy group, being statistically significant in one study; the other only mentioned that IFN-γ was increased in the immunotherapy group and not whether the difference was statistically significant.

TNF-α: In four trials [10], [59], [60], [62] differences in TNF-α levels between immunotherapy and control groups were reported. In one study [60] the level had decreased at the end of 2 months, but detailed data were not given. In another study [62] levels of TNF-α in the supernates of unstimulated polymorphonuclear leucocytes and monocytes fell steeply and significantly (p<0.05) in recipients of *M.vaccae*, whereas they changed minimally in placebo recipients. When the data were combined, statistical difference was lost (P = 0.31), see Table S6.

Immunological factors induced by Th2 lymphocytes: IL-4: two trials [59], [62] reported the level of IL-4 and both of them showed a significant difference between the immunotherapy group and control group (data not shown).The immunotherapy had significant effect of reducing the grossly raised level of IL-4 on admission in newly diagnosed TB patients after one [59], or one, two and three [62] month(s) administration (P<0.001 in both studies), suggesting that the immunotherapy helped to reduce the influence of Th2 to the benefit of the patients.

IL-6: Data from 2 trials [10], [14] were combined, and the results of meta analysis showed that there was no statistical significance between the immunotherapy and control groups (P = 0.52), see Table S6.

#### 3.3 Adverse events

None of the included studies reported any systemic adverse events, however, there were local effects existed, and the most frequently mentioned adverse events were local induration at the injection site [9]–[12], [14], [18], [19], [22], [27]–[29], [31], [33]–[35], [38], [39], [43], [45], [49]–[51], [54]–[56], [58], [60], fever [9]–[12], [14], [18], [19], [31], [33], [43], [45], [50], [51], [54], [55] and sore arm/pain [29], [33], [45], [61].

**Figure 6:**
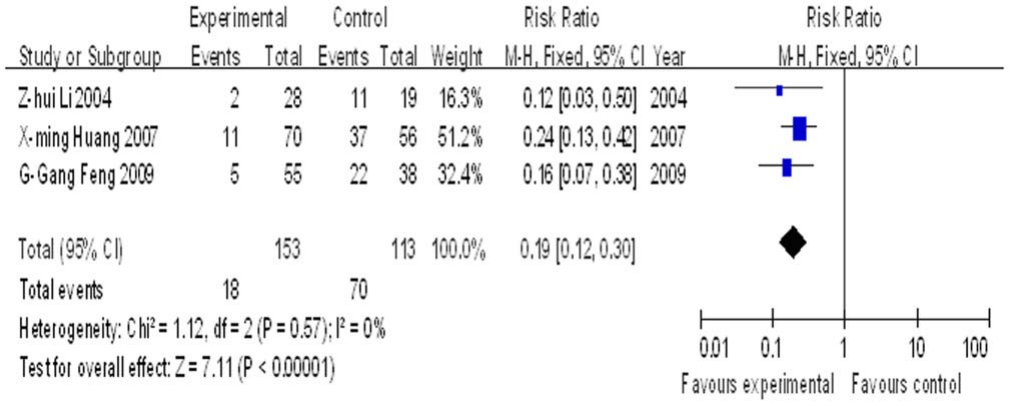
Meta analysis of liver damage rates in TB patients with hepatitis B. Here “Favours control” is put to the right of the line and “Favours experimental” to the left of the line, which is reversed compared to Figures 2, 3, 4, and 5, for the index here is “liver damage rates”, which is a negative indicator, while those in Figures 2, 3, 4, and 5 are positive ones. The “Total” column of each group in each trial is the number of patients who completed the whole course of treatment.

For TB patients co-infected with hepatitis B, the meta analysis of liver damage rates shows that *M.vaccae* has protective effects on liver function (P<0.05)(Figure 6).

## Discussion

Heat-killed *M.vaccae* is a new kind of immune modulator which, in the case of TB, can replace immunopathology with protective antibacterial immunity [58] and enhance immunoregulation through induction of T-regulatory cells [3]. Although anti-TB chemotherapy can kill most of the rapidly replicating bacilli within days, a “persister” population of slowly replicating or dormant organisms needs long-term treatment for at least 6 months [63], and if treatment is stopped early, there is a high probability of relapse. *M.vaccae* can shorten the duration of therapy and induce immune responses that kill the persister organisms [3], [58], accelerate sputum conversion and promote absorption of lesions and healing of cavities. “The guidelines for implementation of Tuberculosis Control Program in China” requires that never-treated TB patients should have repeated sputum smear examinations in their follow-up period at the end of the 2nd, 5th and 6th month. This shows that the overall RRs of smear AFB- rates are greater than one, and with statistical significance, regardless of chemotherapy used.

Although the meta-analysis of sputum culture conversion rates does not show any statistical significance at the end of following up period, the results at 1 and 2 months of follow-up show a significant difference, implying that *M.vaccae* can help to improve sputum conversion to AFB− in patients without complicating medical conditions. An important point to note is that chemotherapy alone is very successful at removing AFB from the third month onwards, giving almost no chance of showing any improvement due to an additional therapy at the later time-points. For TB patients co-affected with diabetes mellitus, hepatitis B or pneumosilicosis, the overall effect does not achieve statistical significance. The results show that the effect of *M.vaccae* is better in TB patients without complicating medical conditions than in those with such conditions. Several factors may account for it: firstly, the total research subjects with complicating medical conditions were much less than those without, which resulted in a false negative estimation; Secondly, the limited subjects were subjected to several different follow-up time points, which further reduced the sample size; Thirdly, the trials targeted at the newly-diagnosed TB patients co-affected with hepatitis B or pneumosilicosis had only results at 3 months or later, which gives almost no chance of showing any bacteriological improvement (see above); And finally, it's really the case and the mechanism is needed to be determined. As for focal changes and cavity closures on X-ray, the results show that *M.vaccae* can help to promote absorption of lesions (both complete and marked absorption) and cavity closure. *M.vaccae* is safe and well tolerated without any systemic adverse events reported in the included studies. A potentially important novel feature reported in this paper is that *M.vaccae* has a protective effect on liver function in those with TB complicated by infectious hepatitis. Is it the true effect, or, just a distorted estimate because of the bias (only three trials with low quality are included)? More studies of high quality are warranted.

The quality assessment showed that in general, the non-Chinese clinical trials had better quality than those carried out in China, but it did not introduce any heterogeneity in sputum culture negative conversion rate, nor did it substract any importance to the overall results.

Our study includes the reports specially targeted at never-treated TB patients, without restriction on language (48 out of 54 were published in Chinese), with a total number of 7149 TB patients involved. The study provides pooled estimates in different groups with and without complicating co-factors. The conclusion of this study is not consistent with study de Bruyn G 2003 [64], which only included studies in English and did not specifically target at never-treated TB patients. There was a review [3] of all studies on immunotherapy with *M.vaccae*, as prepared in England and including some conducted under suboptimal conditions, but it was not a systematic review and merely demonstrated the results of each study without pooled estimates and, furthermore, it did not target specially at never-treated TB patients.

There are several limitations of our study: first, the follow-up period was not long enough to obtain the results of long-term effects, for example, recurrence, drug resistance, and so on, and the follow-up time points varied across studies. Secondly, immunological characteristics were only determined in a minority of the studies so that the effect of M.vaccae on Th1/Th2 levels in TB remains to be further confirmed and thirdly, the multi-dose use of M.vaccae in all trials conducted in China was not standardised, so the optimum dosing schedule has still to be determined.

Conclusion: *M.vaccae* is a well-tolerated and helpful addition to treatment for never-treated TB patients in terms of significantly improved sputum conversion and radiological appearances.

## Supporting Information

Table S1 **Characteristics of included studies.** MV: Mycobacterium vaccae H: Isoniazid R: Rifampicin Z: Pyrazinamide E: Ethambutol S: Streptomycin L: Levofloxacin Re: Rifapentine P: Pyridoxine. The strains of Mycobacterium tuberculosis were 100% susceptible to drugs of at least H and R in trial with the reference No. 9, 10, 28, 29, 42, 58, 59, 62, and 4% resistant to both H and R (MDR) in trial with the reference No. 57; other studies not mentioned the drug-susceptibility patterns.
(DOC)

Table S2 **Quality assessment of included studies.** Y: yes; N: no; U: unclear.
(DOC)

Table S3 **Meta analysis of AFB^−^ rates for TB patients at different follow-up time points.** #: N_E_ means the subject number of intervention group, N_C_ means the subject number of control group. ▴: F = Fixed model, R = Random model. *: P_H_ means the p value of heterogeneity test (α = 0.05).
(DOC)

Table S4 **Meta analysis of focal change on X-ray chest film.** #: N_E_ means the subject number of intervention group, N_C_ means the subject number of control group. ▴: F = Fixed model, R = Random model. *: P_H_ means the p value of heterogeneity test (α = 0.05).
(DOC)

Table S5 **Meta analysis of cavity closure rates.** #: N_E_ means the subject number of intervention group, N_C_ means the subject number of control group. ▴: F = Fixed model, R = Random model. *: P_H_ means the p value of heterogeneity test (α = 0.05).
(DOC)

Table S6 **Meta analysis of the levels of Th1/Th2 indicators.** #: N_E_ means the subject number of intervention group, N_C_ means the subject number of control group. ▴: F = Fixed model, R = Random model. *: P_H_ means the p value of heterogeneity test (α = 0.05).
(DOC)
