# Supplementary material for: Mycobacterium vaccae as Adjuvant Therapy to Anti-Tuberculosis Chemotherapy in Never-Treated Tuberculosis Patients: A Meta-Analysis
Source: PLoS One. 2011 Sep 6;6(9):e23826. doi: 10.1371/journal.pone.0023826 (PMC3167806; doi:10.1371/journal.pone.0023826)
Supplement: Table S1 — Characteristics of included studies. MV: Mycobacterium vaccae H: Isoniazid R: Rifampicin Z: Pyrazinamide E: Ethambutol S: Streptomycin L: Levofloxacin Re: Rifapentine P: Pyridoxine. The strains of Mycobacterium tuberculosis were 100% susceptible to drugs of at least H and R in trial with the reference No. 9, 10, 28, 29, 42, 58, 59, 62, and 4% resistant to both H and R (MDR) in trial with the reference No. 57; other studies not mentioned the drug-susceptibility patterns. (DOC) [file pone.0023826.s001.doc]

Table S1 Characteristics of included studies

| NO. | Author | Nation | Age | Combined disease | Number | MV administration | Experiment group | | | | Control group | |
| --- | --- | --- | --- | --- | --- | --- | --- | --- | --- | --- | --- | --- |
|  |  |  |  |  |  |  | Number | Anti-tuberculosis treatment regimen △ | MV doses | MV administration starting time | Number | Anti-tuberculosis treatment regimen △ |
| 1 | S-hua Lu 1998 [9] | China | (37.8±13.7)/(43.2±10.6) | - | 142 | multi-doses | 71 | 2HRZE(S) / 2HR+MV | 0.1 mg l MV *1st，0.5mg per3-4weeks，for 6 month | at end of 2^nd^ week | 71 | 2HRZE(S) / 4HR |
| 2 | Wei Wang 1999 [10] | China | 18-63(40±14)/20-65(38±14) | - | 70 | multi-doses | 35 | 2HRZS/4HR+MV | 0.1mg/1^st^，0.5mg per 2 weeks | at end of 2^nd^ week | 35 | 2HRZS/4HR |
| 3 | S-hua Yang 2001 [11] | China | (15-65)33.2 | - | 82 | multi-doses | 41 | 2HRZE/4HR+MV | 0.5mg per month | at end of 2^nd^ week | 41 | 2HRZE/4HR |
| 4 | Y-ai Luo 2001 [12] | China | 18-65 | - | 342 | multi-doses | 171 | 2HRZE/2HR+MV | 0.1mg MV *1st，0.5mg per3-4weeks，for 6 months | at end of 2^nd^ week | 171 | 2HRZE/ 4HR |
| 5 | Li Wen 2003 [13] | China | 39/41 | - | 90 | multi-doses | 45 | 2H_3_R_3_Z_3_E_3_/4H_3_R_3_+MV | a dose of 22.5ug per 10 days for 3months | From 1^st^ day | 45 | 2H_3_R_3_Z_3_E_3_/4H_3_R_3_ |
| 6 | G-qing Zhang 2004 [14] | China | (40±14)/(39±14) | - | 80 | multi-doses | 40 | 2HRZE/4HR+MV | a dose of 22.5ug per 2 weeks，for 6 months | at end of 2^nd^ week | 40 | 2HRZE/4HR |
| 7 | H-sheng Zhou 2004 [15] | China | (16-86)45.6 | - | 256 | multi-doses | 128 | 2HRZE/4HR+MV | a dose of 22.5ug per week for 3 months | - | 128 | 2HRZE/4HR |
| 8 | Lin Luo 2004 [16] | China | 15-65 | - | 76 | multi-doses | 38 | 2HRZE/2HR+MV | 0.1mg/次*1st，0.5mg per 2 weeks，for 6 months | From 1^st^ day | 38 | 2HRZE / 4HR |
| 9 | M-ling Hao 2004 [17] | China | (17-68)42.4/(19-65)41.8 | - | 90 | multi-doses | 45 | 2HRZE/4HR+MV | a dose of 22.5ug per 2 weeks for 3 months | at end of 2^nd^ week | 45 | 2HRZE/4HR |
| 10 | W-hong Gao 2004 [18] | China | 15-70 | - | 168 | multi-doses | 84 | 2HRZE/4HR+MV | a dose of 22.5ug per week for 2 months | at end of 1^st^ week | 84 | 2HRZE/4HR |
| 11 | Yong Peng 2004 [19] | China | 18-60 | - | 40 | multi-doses | 20 | 2H_3_R_3_Z_3_S_3_/4H_3_R_3_+MV | a dose of 22.5ug per 2 weeks，for 6 months | at end of 1^st^ week | 20 | 2H_3_R_3_Z_3_S_3_/4H_3_R_3_ |
| 12 | G-xing Liu 2005 [20] | China | 36.5/36.2 | - | 208 | multi-doses | 104 | 2H_3_R_3_ E_3_Z_3_/4H_3_R_3_+MV | 0.5mg MV+0.1ml saline per 3 weeks | At end of 10^th^ day | 104 | 2H_3_R_3_ E_3_Z_3_/4H_3_R_3_ |
| 13 | W-hui Fan 2005 [21] | China | (30-77)61.6/(31-75)60.5 | - | 120 | multi-doses | 60 | 2HRZE/4HRE+MV | a dose of 22.5ug per 2 weeks，for 6 times | - | 60 | 2HRZE/4HRE |
| 14 | Y-long Li 2005 [22] | China | (19-60)/(18-62) | - | 169 | multi-doses | 85 | 2HRZS(E) / 4HR +MV | a dose of 22.5ug per week | - | 84 | 2HRZS(E) / 4HR+ |
| 15 | Hong Li 2006 [23] | China | (16-81)26.73/(16-75)17.88 | - | 109 | multi-doses | 58 | 2HRZE(S)+MV / 4HR+MV | a dose of 22.5ug per week, for 6 months | At 1^st^ day | 51 | 2HRZE(S) / 4HR |
| 16 | S-yin Mu 2006 [24] | China | - | - | 80 | multi-doses | 40 | 2HRZEL/4HR+MV | A dose of 22ug per 2 weeks for 6 months | - | 40 | 2HRZE/ 4HR |
| 17 | S-yuan Hu 2006 [25] | China | 18-59 | - | 179 | multi-doses | 92 | 2H_3_R_3_S_3_ ( E_3_) Z_3_/ 4H_3_R_3_+MV | a dose of 22.5ug per 15 days for 3 months | - | 87 | 2HRZE/4HR |
| 18 | X-yuan Xu 2006 [26] | China | (18-62)/(19-61) | - | 198 | multi-doses | 99 | 2H_3_R_3_Z_3_E_3_/4H_3_R_3_+MV | a dose of 0.5mg per 2 weeks for 6 months | at end of 2^nd^ week | 99 | 2H_3_R_3_Z_3_E_3_/4H_3_R_3_ |
| 19 | Y-mei Chen 2006 [27] | China | (21-72)46.5/(20-70)45 | - | 116 | multi-doses | 58 | 2HRZE/4HR+MV | 22.5ug per 2 weeks for 11 times | at end of 2^nd^ week | 58 | 2HRZE/4HR |
| 20 | Hui Fan 2007 [28] | China | 46.2 | - | 120 | multi-doses | 60 | 2H_3_R_3_Z_3_S_3_/4H_3_R_3_+MV | a dose of 22.5ug per 2 weeks | - | 60 | 2H_3_R_3_Z_3_S_3_/4H_3_R_3_ |
| 21 | Y-juan Tian 2007 [29] | China | 18-65 | - | 128 | multi-doses | 64 | 2HRZS(E)/4HR+MV | a dose of 22.5ug per 10 days for 2 months, then a dose of 22.5ug per 15 days for 4 months | - | 64 | 2HRZS(E)/4HR |
| 22 | Y-xiang Zhang 2007 [30] | China | 56/57 | - | 284 | multi-doses | 121 | 2H_3_R_3_Z_3_E_3_/4H_3_R_3_+MV | a dose of 22.5ug per 15 days for 6 months | - | 163 | 2H_3_R_3_Z_3_E_3_/4H_3_R_3_ |
| 23 | C-yun Wang 2008 [31] | China | (16-68)48.5/(18-70)47.9 | - | 126 | multi-doses | 63 | 2HL_2_ZE(S)/4HL_2_+MV | a dose of 22.5ug per week for 2 months | at end of 1^st^ week | 63 | 2HL_2_ZE(S)/4HL_2_ |
| 24 | J-biao Yang 2008 [32] | China | 17-68 | - | 102 | multi-doses | 51 | 2H_3_R_3_Z_3_E_3_(S_3_)/4H_3_R_3_+MV | 22.5ug*8(4 per 15days and 4 per 20-30 days later) | at end of 1^st^ week | 51 | 2H_3_R_3_Z_3_E_3_(S_3_)/4H_3_R_3_ |
| 25 | Ming Chen 2008 [33] | China | (16-72)35.9 | - | 90 | multi-doses | 45 | 2HRZS(E)/4HR+MV | a dose of 22.5ug per week | Pre-8 weeks | 45 | 2HRZS(E)/4HR |
| 26 | T-xuan Lu 2008 [34] | China | (16-60)42.4/(16-58)41.8 | - | 98 | multi-doses | 49 | 2H_3_R_3_Z_3_E_3_/4H_3_R_3_+MV | a dose of 22.5ug per 2 weeks for 3 months | at end of 1^st^ week | 49 | 2H_3_R_3_Z_3_E _3_/4H_3_R_3_ |
| 27 | W-qiang Zhang 2008 [35] | China | (19-60)/(18-62) | - | 169 | multi-doses | 85 | 2HRZS ( E) /4HR+MV | a dose of 22.5ug per week | From 1^st^ day | 84 | 2HRZS( E) /4HR |
| 28 | X-fang Li 2008 [36] | China | 18-65 | - | 95 | multi-doses | 47 | 2H_3_R_3_Z_3_E_3_/4H_3_R_3_+MV | a dose of 22.5ug per week | - | 48 | 2H_3_R_3_Z_3_E_3_/4H_3_R_3_ |
| 29 | Z-di Sun 2008 [37] | China | 38.5/37.8 | - | 58 | multi-doses | 26 | 3HRZE/9HR+MV | a dose of 22.5ug per 2 weeks for 6 times | at end of 2^nd^ week | 32 | 3HRZE/9HR |
| 30 | Q-rui Song 2009 [38] | China | (21-68)49.2 | - | 58 | multi-doses | 35 | 2H_3_R_3_Z_3_E_3_/4H_3_R_3_+MV | a dose of 22.5ug per 2 weeks for 6 months | - | 23 | 2H_3_R_3_Z_3_E_3_/4H_3_R_3_ |
| 31 | Hong-Zhu 2005 [39] | China | 20-67 | - | 96 | multi-doses | 49 | 2HREZ(S) / 4HR+MV | a dose of 22.5ug per 2 weeks | at end of 2^nd^ week | 47 | 2HREZ(S) / 4HR |
|  |  |  |  | diabetes | 71 | multi-doses | 36 | 2HREZ(S) / 4HR +MV+ insulin / Hypoglycemic agents | a dose of 22.5ug per 2 weeks | at end of 2^nd^ week | 35 | 2HREZ(S) / 4HR + insulin / Hypoglycemic agents |
| 32 | J-zhen Xie 2005 [40] | China | 46.5/45.8 | diabetes | 40 | multi-doses | 20 | 2HRZE/4HR+MV+ insulin / Hypoglycemic agents | a dose of 22.5ug per week for 2 months | From 1^st^ day | 20 | 2HRZE/4HR+ insulin / Hypoglycemic agents |
| 33 | X-li Yuan 2005 [41] | China | (32-75)42.5 | diabetes | 160 | multi-doses | 80 | 3S(E) HRZ/ 9HR +MV +insulin / Hypoglycemic agents | a dose of 22.5ug per week for 3 months | - | 80 | 3S(E) HRZ/ 9HR+insulin / Hypoglycemic agents |
| 34 | Z-qing He 2005 [42] | China | 44.8 | diabetes | 52 | multi-doses | 26 | 2H_3_R_3_Z_3_S_3_ ( E_3_) / 4H_3_R_3_+MV+ insulin / Hypoglycemic agents | a dose of 22.5ug per 2 weeks for 6 months | at end of 1^st^ week | 26 | 2H_3_R_3_Z3S_3_ ( E_3_) / 4H_3_R_3_+ insulin / Hypoglycemic agents |
| 35 | W-ming Shen2006 [43] | China | 18-60 | diabetes | 96 | multi-doses | 50 | 2H_3_R_3_Z_3_E_3_/4H_3_R_3_+MV +insulin / Hypoglycemic agents | a dose of 22.5ug per week for 6 months | at end of 2^nd^ week | 46 | 2H_3_R_3_Z_3_E_3_/4H_3_R_3_ +insulin / Hypoglycemic agents |
| 36 | Wei Ouyang 2008 [44] | China | 46.5/44.2 | diabetes | 43 | multi-doses | 23 | E+H+R+insulin / Hypoglycemic agents+ Traditional medicine +MV | 0.1mg 1^st^,0.5mg per 3 weeks later for 3 months | - | 20 | E+H+R+insulin / Hypoglycemic agents+ Traditional medicine |
| 37 | Y-liang Wang 2009 [45] | China | (34-75)/(35-74) | diabetes | 120 | multi-doses | 60 | 3HRZE/9HR+MV+Hypoglycemic agent/ insulin | a dose of 22.5ug per 15 days*pre-3 months, a dose of 22.5ug per month for 3 months later | - | 60 | 3HRZE/9HR+Hypoglycemic agent/ insulin |
| 38 | Z-hui Li 2004 [46] | China | (18-60)41.5/(18-62)43.2 | HBsAg+ | 60 | multi-doses | 30 | 2HRZE(S) / 2HR+ glucurone +MV | a dose of 22.5ug two times per week for 6 months | - | 30 | 2HRZE(S) / 4HR+ glucurone |
| 39 | X-ming Huang 2007 [47] | China | (18-70)42.6 | HBsAg+ | 126 | multi-doses | 70 | 2HERS(Z)/4HR+MV | a dose of 22.5ug per 2 weeks for 6 months | - | 56 | 2HERS(Z)/4HR |
| 40 | G-gang Feng 2009 [48] | China | (18-60)41.5/(18-62)43.2 | HBsAg+ | 120 | multi-doses | 60 | 2HRZE/2HR+ glucurone +MV | a dose of 22.5ug per 2 weeks for 6 months | - | 60 | 2HRZE/4HR+ glucurone |
| 41 | Jia Shi 2005 [49] | China | 62/60 | pneumonoconiosis | 60 | multi-doses | 30 | 3HRZE/6HR | a dose of 22.5ug per 3-4 weeks for 6 months | at end of 2^nd^week | 30 | 3HRZE /6HR |
| 42 | Xia Xu 2008 [50] | China | 63-75(68) | pneumonoconiosis | 104 | multi-doses | 53 | 3HRZE/6HRE/3HR+MV | a dose of 22.5ug per 2 weeks for 6 months | at end of 2^nd^ week | 51 | 3HRZE/6HRE/3HR |
| 43 | F-jing Meng 2005 [51] | China | (60-86)67/(60-87)66 | elder | 120 | multi-doses | 60 | 2HRZE/ 4HR+MV | a dose of 22.5ug per week for 2 months | - | 60 | 2HRZE/ 4HR |
| 44 | Zhong Li 2005 [52] | China | (63-82)67.2/(60-93)66.8 | elder | 52 | multi-doses | 25 | 2HRZE(S) / 4HR+MV | a dose of 22.5ug per 2 weeks,for 2 months | - | 27 | 2HRZE(S) / 4HR |
| 45 | J-hua Guo 2006 [53] | China | (68.7±10.1)/(67.6±9.65) | elder | 88 | multi-doses | 58 | 2HRZE/4HRE+MV | a dose of 22.5ug per 10 days for 6 months | From 1^st^ day | 30 | 2HRZE/4HR |
| 46 | Bo Wang 2007 [54] | China | 68±5/69±4 | elder | 60 | multi-doses | 30 | 2HREZ/4HR+MV | a dose of 22.5ug per 2 weeks for 3 months | at end of 2^nd^ week | 30 | 2HREZ/4HR |
| 47 | S-ping Yang 2008 [55] | China | (68±5)/(69±4) | elder | 60 | multi-doses | 30 | 2HRZE/4HR+MV | a dose of 22.5ug per 2 weeks for 3 months | at end of 2^nd^ week | 30 | 2HRZE/4HR |
| 48 | Bo Chen 2009 [56] | China | 65-85 | elder | 121 | multi-doses | 61 | 2HReZE/4HRe+MV | a dose of 22.5ug per 2 weeks for 6 months | at end of 2^nd^ week | 60 | 2HReZE/4HRe |
| 49 | Corlan 1997 [57] | Romania | 16-80 | - | 206 | single-dose | 97 | 2HRZS_2_/4HR_2_ +MV | strain NCTC 11659,batch A5 (0.1 ml, 10 mg/ml; approximately 10^9^ bacilli) | 1 month after the first course of chemotherapy was started | 109 | 2HRZS_2_/4HR_2_+injection of saline |
| 50 | DITG 1999 [58] | South Africa | 18-65 | 34% being HIV+ | 347 | single-dose | 172 | 2HRZEP/4H_3_R_3_P+MV | 0.1 ml, 10^9^ MV | On day 8 | 175 | 2HRZEP/4H_3_R_3_P +saline |
| 51 | Dlugovitzky 1999 [59] | South American | 40 (mean) | HIV - | 40 | single-dose | 23 | 2HRS(Z)/4HR+MV | NCTC 11659 (SRL 172) batch A4, equivalent to 10^9^ bacilli per dose | 15 days after/1^st^ day | 17 | 2HRS(Z)/4HR+ tetanus toxoid/physiological saline |
| 52 | Johnson 2000 [60] | Uganda | 18-50 | HIV - | 120 | single-dose | 61 | 2HRZE/4HR+MV | NCTC 11659, 0.1 ml containing 10^9^ organisms in sterile borate buffered saline | On the 8^th^ day | 59 | 2HRZE/4HR+ placebo 0.1 ml of sterile borate-buffered saline |
| 53 | Mwinga 2002 [61] | Zambia | 18-60 | HIV+ | 760 | single-dose | 374 | 2HRZE/6HE+MV  2HRZS/6HE+MV | 0.1 ml SRL172 containing 10^9^ heat-killed organisms (M vaccae strain NCTC11659) in sterile borate-buffered saline | Within first 2 weeks | 386 | 2HRZE(S)/6HE+ placebo (0.1 ml sterile borate-buffered saline) |
| 54 | Dlugovitzky 2006 [62] | Argentina | (33.1±14.8)/(36.8±12.8) | HIV- | 22 | triple-doses | 12 | 2HRZE/4HR+MV | strain NCTC 11659, suspended in 1.0 ml of boratebuffered (pH 8) physiological saline | On days 1, 30 and 60 of chemotherapy | 10 | 2HRZE/4HR+placebo (buffer alone) |

MV: Mycobacterium vaccae H: Isoniazid R: Rifampicin Z: [Pyrazinamide](app:ds:pyrazinamide) E: Ethambutol S: Streptomycin L: Levofloxacin Re: Rifapentine P: Pyridoxine

△: The strains of M. tuberculosis were 100% susceptible to drugs of at least H and R in trial with the reference No. 9, 10, 28, 29, 42, 58, 59, 62, and 4% resistant to both H and R (MDR) in trial with the reference No. 57; other studies not mentioned the drug-susceptibility patterns.
