# Supplementary material for: Mycobacterium vaccae as Adjuvant Therapy to Anti-Tuberculosis Chemotherapy in Never-Treated Tuberculosis Patients: A Meta-Analysis
Source: PLoS One. 2011 Sep 6;6(9):e23826. doi: 10.1371/journal.pone.0023826 (PMC3167806; doi:10.1371/journal.pone.0023826)
Supplement: Table S2 — Quality assessment of included studies. Y: yes; N: no; U: unclear. (DOC) [file pone.0023826.s002.doc]

Table S2 Quality assessment of included studies

| NO. | Included studies | Type of study | Adequate sequence generation | Allocation concealment | Blinding | Incomplete outcome data addressed |
| --- | --- | --- | --- | --- | --- | --- |
| 1 | S-hua Lu 1998 [9] | CCT | U | N | U | N |
| 2 | Wei-Wang 1999 [10] | CCT | U | N | U | N |
| 3 | S-hua Yang 2001 [11] | CCT | U | N | U | N |
| 4 | Y-ai Luo 2001 [12] | CCT | U | N | U | N |
| 5 | Li-Wen 2003 [13] | CCT | U | N | U | N |
| 6 | G-qing Zhang 2004 [14] | CCT | U | N | U | N |
| 7 | H-sheng Zhou 2004 [15] | CCT | U | N | U | N |
| 8 | Lin-Luo 2004 [16] | CCT | U | N | U | N |
| 9 | M-ling Hao 2004 [17] | CCT | U | N | U | N |
| 10 | W-hong Gao 2004 [18] | CCT | U | N | U | N |
| 11 | Yong-Peng 2004 [19] | CCT | U | N | U | N |
| 12 | Z-hui Li 2004 [46] | CCT | U | N | U | N |
| 13 | F-jing Meng 2005 [51] | CCT | U | N | U | N |
| 14 | G-xing Liu 2005 [20] | CCT | U | N | U | N |
| 15 | Hong-Zhu 2005 [39] | CCT | U | N | U | N |
| 16 | Jia Shi 2005 [49] | RCT | Y | N | N | Y |
| 17 | J-zhen Xie 2005 [40] | CCT | U | N | U | N |
| 18 | W-hui Fan 2005 [21] | CCT | U | N | U | N |
| 19 | X-li Yuan 2005 [41] | CCT | U | N | U | N |
| 20 | Y-long Li 2005 [22] | CCT | U | N | U | N |
| 21 | Zhong Li 2005 [52] | CCT | U | N | U | N |
| 22 | Z-qing He 2005 [42] | CCT | U | N | U | N |
| 23 | Hong Li 2006 [23] | CCT | U | N | U | N |
| 24 | J-hua Guo 2006 [53] | CCT | U | N | U | N |
| 25 | W-ming Shen 2006 [43] | CCT | U | N | U | N |
| 26 | S-yin Mu 2006 [24] | CCT | U | N | U | N |
| 27 | S-yuan Hu 2006 [25] | CCT | U | N | U | N |
| 28 | X-yuan Xu 2006 [26] | CCT | U | N | U | N |
| 29 | Y-mei Chen 2006 [27] | CCT | U | N | U | N |
| 30 | Bo Wang 2007 [54] | CCT | U | N | U | N |
| 31 | Hui Fan 2007 [28] | CCT | U | N | U | N |
| 32 | X-ming Huang 2007 [47] | CCT | U | N | U | N |
| 33 | Y-juan Tian 2007 [29] | CCT | U | N | U | N |
| 34 | Y-xiang Zhang 2007 [30] | CCT | U | N | U | N |
| 35 | C-yun Wang 2008 [31] | CCT | U | N | U | N |
| 36 | J-biao Yang 2008 [32] | RCT | Y | N | N | Y |
| 37 | Ming Chen 2008 [33] | CCT | U | N | U | N |
| 38 | S-ping Yang 2008 [55] | CCT | U | N | U | N |
| 39 | T-xuan Lu 2008 [34] | CCT | U | N | U | N |
| 40 | Wei Ouyang 2008 [44] | CCT | U | N | U | N |
| 41 | W-qiang Zhang 2008 [35] | CCT | U | N | U | N |
| 42 | X-fang Li 2008 [36] | CCT | U | N | U | N |
| 43 | Xia Xu 2008 [50] | CCT | U | N | U | N |
| 44 | Z-di Sun 2008 [37] | CCT | U | N | U | N |
| 45 | Bo Chen 2009 [56] | CCT | U | N | U | N |
| 46 | G-gang Feng 2009 [48] | CCT | N | N | N | Y |
| 47 | Q-rui Song 2009 [38] | CCT | U | N | U | N |
| 48 | Y-liang Wang 2009 [45] | RCT | Y | N | Y | Y |
| 49 | Corlan 1997 [57] | RCT | Y | N | Y | Y |
| 50 | DITG 1999 [58] | RCT | Y | Y | Y | Y |
| 51 | Dlugovitzky 1999 [59] | CCT | N | N | U | N |
| 52 | Johnson 2000 [60] | RCT | Y | Y | Y | Y |
| 53 | Mwinga 2002 [61] | RCT | Y | Y | Y | Y |
| 54 | Dlugovitzky 2006 [62] | RCT | Y | U | Y | N |

Y: yes; N: no; U: unclear
