# Supplementary material for: Mycobacterium vaccae as Adjuvant Therapy to Anti-Tuberculosis Chemotherapy in Never-Treated Tuberculosis Patients: A Meta-Analysis
Source: PLoS One. 2011 Sep 6;6(9):e23826. doi: 10.1371/journal.pone.0023826 (PMC3167806; doi:10.1371/journal.pone.0023826)
Supplement: Table S3 — Meta analysis of AFB− rates for TB patients at different follow-up time points. #: NE means the subject number of intervention group, NC means the subject number of control group. ▴: F = Fixed model, R = Random model. *: PH means the p value of heterogeneity test (α = 0.05). (DOC) [file pone.0023826.s003.doc]

Table S3 Meta analysis of AFB^-^ rates for TB patients at different follow-up time points

| Subjects | 1 month | | | | | | 2 months | | | | | | 3 months | | | | | |
| --- | --- | --- | --- | --- | --- | --- | --- | --- | --- | --- | --- | --- | --- | --- | --- | --- | --- | --- |
|  | Studies | N_E_/N_C_ ^#^ | Model▲ | P_H_^*^ | Pooled RR(95%CI) | P | Studies | N_E_/N_C_ | Model | P_H_ | Pooled RR (95%CI) | P | Studies | N_E_/N_C_ | Model | P_H_ | Pooled RR (95%CI) | P |
| TB (smear AFB^-^) | 12 | 531/543 | F | 0.99 | 1.64  (1.43,1.89) | <0.00001 | 21 | 1241/1270 | F | 0.18 | 1.26  (1.09,1.56) | <0.00001 | 15 | 981/983 | R | 0.003 | 1.12  (1.06,1.18) | <0.0001 |
| TB(culture AFB^-^) | 5 | 360/357 | F | 0.28 | 2.28  (1.80,2.90) | <0.00001 | 6 | 457/464 | F | 0.50 | 1.25  (1.16,1.34) | <0.00001 | - | - | - | - | - | - |
| TB +diabetes  (smear AFB-) | 3 | 163/160 | R | 0.006 | 1.70  (0.94,3.06) | 0.08 | 5 | 239/232 | R | 0.0002 | 1.38  (1.08,1.77) | 0.009 | 5 | 225/221 | F | 0.20 | 1.27  (1.12,1.44) | 0.0001 |
| TB+ HBsAg^+^  (smear AFB-) | - | - | - | - | - | - | - | - | - | - | - | - | - | - | - | - | - | - |
| TB+pneumosilicosis  (smear AFB-) | - | - | - | - | - | - | - | - | - | - | - | - | 2 | 83/81 | R | <0.00001 | 1.46  (0.21,10.06) | 0.70 |
| elderly TB (smear AFB-) | 2 | 121/120 | F | 0.12 | 1.69  (1.33,2.14) | <0.0001 | 5 | 239/210 | F | 0.68 | 1.44  (1.26,1.65) | <0.00001 | 2 | 118/90 | R | 0.002 | 1.19  (0,62,2.26) | 0.61 |
| subjects | 4 months | | | | | | 5 months | | | | | | 6 months | | | | | |
|  | Studies | N_E_/N_C_ | model | P_H_ | Pooled RR(95%CI) | P | Studies | N_E_/N_C_ | model | P_H_ | Pooled RR (95%CI) | P | Studies | N_E_/N_C_ | model | P_H_ | Pooled RR (95%CI) | P |
| TB(smear AFB-) | 9 | 544/542 | R | 0.0002 | 1.07  (1.00,1.14) | 0.05 | 8 | 546/546 | F | 0.19 | 1.03  (1.00,1.05) | 0.04 | 26 | 1812/1857 | R | <0.00001 | 1.06  (1.03,1.10) | <0.0001 |
| TB(culture AFB-) | 6 | 397/392 | F | 0.54 | 1.00  (0.96,1.05) | 0.87 | - | - | - | - | - | - | 7 | 659/685 | F | 0.15 | 1.03  (0.99,107) | 0.12 |
| TB +diabetes  (smear AFB-) | - | - | - | - | - | - | - | - | - | - | - | - | 3 | 112/107 | F | 0.55 | 1.14  (1.03,1.26) | 0.01 |
| TB+ HBsAg^+^  (smear AFB-) | - | - | - | - | - | - | - | - | - | - | - | - | 2 | 83/57 | F | 0.99 | 1.02  (0.94,1.10) | 0.65 |
| TB+pneumosilicosis  (smear AFB-) | - | - | - | - | - | - | - | - | - | - | -- | - | - | - | - | - | - | - |
| elderly TB (smear AFB-) | 2 | 60/60 | F | 0.78 | 1.38  (1.10,1.73) | 0.005 | - | - | - | - | - | - | 4 | 212/183 | F | 0.13 | 1.21  (1.10,1.32) | <0.0001 |

#: N_E_ means the subject number of intervention group, N_C_ means the subject number of control group.

▲: F=Fixed model, R=Random model

*: P_H_ means the p value of heterogeneity test (α=0.05)
