# Supplementary material for: Mycobacterium vaccae as Adjuvant Therapy to Anti-Tuberculosis Chemotherapy in Never-Treated Tuberculosis Patients: A Meta-Analysis
Source: PLoS One. 2011 Sep 6;6(9):e23826. doi: 10.1371/journal.pone.0023826 (PMC3167806; doi:10.1371/journal.pone.0023826)
Supplement: Table S4 — Meta analysis of focal change on X-ray chest film. #: NE means the subject number of intervention group, NC means the subject number of control group. ▴: F = Fixed model, R = Random model. *: PH means the p value of heterogeneity test (α = 0.05). (DOC) [file pone.0023826.s004.doc]

Table S4 Meta analysis of focal change on X-ray chest film

| Subjects | Focal change | 2 months | | | | | | | 4 months | | | | | | 6 months | | | | | |
| --- | --- | --- | --- | --- | --- | --- | --- | --- | --- | --- | --- | --- | --- | --- | --- | --- | --- | --- | --- | --- |
|  |  | Studies | N_E_/N_C_ ^#^ | Model  ▲ | P_H_^*^ | Pooled RR  95%CI | | P | Studies | N_E_/N_C_ | Model | P_H_ | Pooled RR  95%CI | P | Studies | N_E_/N_C_ | Model | P_H_ | Pooled RR  95%CI | P |
| TB | complete absorption | 3 | 295/295 | F | 0.84 | 10.33(1.95,54.78) | | 0.006 | 3 | 295/295 | F | 0.89 | 7.67(2.33,25.20) | 0.0008 | 6 | 578/620 | F | 0.71 | 3.05(2.24,4.16) | <0.00001 |
|  | marked absorption | 11 | 812/847 | F | 0.57 | 1.93(1.64,2.28) | | <0.00001 | 6 | 540/582 | F | 0.98 | 1.54(1.37,1.74) | <0.00001 | 21 | 1686/1657 | R | 0.006 | 1.15(1.08,1.23) | <0.00001 |
|  | absorption | 13 | 843/836 | R | <0.0001 | 1.04(0.87,1.25) | | 0.66 | 7 | 504/504 | R | <0.00001 | 0.86(0.54,1.37) | 0.53 | 22 | 1643/1636 | R | <0.00001 | 0.90(079,1.02) | 0.02 |
|  | unchanged | 8 | 633/626 | R | 0.006 | 0.78(0.44,1.37) | | 0.38 | 4 | 399/399 | F | 0.99 | 0.43(0.17,1.10) | 0.08 | 19 | 1470/1502 | R | 0.0003 | 0.55(0.36,0.85) | 0.007 |
|  | deterioration | 8 | 589/582 | F | 0.31 | 0.38(0.09,1.62) | | 0.19 | 5 | 419/419 | F | 0.61 | 1.86(0.52,6.69) | 0.34 | 15 | 1063/1053 | F | 1.00 | 0.40(0.19,0.85) | 0.02 |
| TB+diabetes | marked absorption | - | - | - | - | - | | - | - | - | - | - | - | - | 4 | 172/167 | F | 0.63 | 0.14(0.04,0.24) | 0.005 |
|  | absorption | - | - | - | - | - | | - | - | - | - | - | - | - | 3 | 176/175 | F | 0.41 | 1.25(1.02,1.53) | 0.03 |
|  | unchanged | - | - | - | - | - | | - | - | - | - | - | - | - | 2 | 96/95 | F | 0.86 | 0.43(0.23,0.78) | 0.005 |
|  | deterioration | - | - | - | - | - | | - | - | - | - | - | - | - | 2 | 96/95 | - | - | 0.19(0.02,1.58) | 0.13 |
| TB+ HBsAg^+^ | marked absorption | - |  | - |  | - | | - | - | - | - | - | - | - | 2 | 83/74 | F | 0.11 | 1.60(1.27,2.01) | <0.0001 |
|  | absorption | - | - | - | - | - | | - | - | - | - | - | - | - | 2 | 83/57 | F | 0.98 | 0.92(0.41,2.03) | 0.83 |
| elderly TB | marked absorption | 3 | 121/120 | F | 0.77 | 2.48(1.58,3.89) | | <0.0001 | 2 | 60/60 | F | 1.00 | 2.25(1.41,3.59) | 0.0007 | 4 | 114/153 | F | 0.32 | 1.47(1.19,1.82) | 0.0003 |
|  | absorption | 3 | 120/120 | F | 0.47 | 1.53(1.04,2.25) | | 0.03 | 3 | 120/120 | F | 0.71 | 1.39(1.07,1.80) | 0.01 | 5 | 211/183 | F | 0.93 | 1.25(1.07,1.47) | 0.006 |
|  | unchanged | 3 | 121/120 | F | 0.93 | 0.46(0.30,0.72) | | 0.0006 | 2 | 60/60 | F | 0.80 | 0.47(0.30,0.74) | 0.001 | 4 | 126/126 | F | 0.71 | 0.36(0.22,0.58) | <0.0001 |
|  | deterioration | - |  | - |  | - | - | - | 2 | 60/60 | F | 1.00 | 0.2(0.02,1.66) | 0.14 | 2 | 60/60 | F | 1.00 | 0.50(0.10,2.63) | 0.41 |

#: N_E_ means the subject number of intervention group, N_C_ means the subject number of control group.

▲: F=Fixed model, R=Random model

*: P_H_ means the p value of heterogeneity test (α=0.05)
