# Supplementary material for: Mycobacterium vaccae as Adjuvant Therapy to Anti-Tuberculosis Chemotherapy in Never-Treated Tuberculosis Patients: A Meta-Analysis
Source: PLoS One. 2011 Sep 6;6(9):e23826. doi: 10.1371/journal.pone.0023826 (PMC3167806; doi:10.1371/journal.pone.0023826)
Supplement: Table S5 — Meta analysis of cavity closure rates. #: NE means the subject number of intervention group, NC means the subject number of control group. ▴: F = Fixed model, R = Random model. *: PH means the p value of heterogeneity test (α = 0.05). (DOC) [file pone.0023826.s005.doc]

Table S5 Meta analysis of cavity closure rates

| Subjects | Cavity closure | 2 months | | | | | | 4 months | | | | | | 6 months | | | | | |
| --- | --- | --- | --- | --- | --- | --- | --- | --- | --- | --- | --- | --- | --- | --- | --- | --- | --- | --- | --- |
|  |  | Studies | N_E_/N_C_ ^#^ | Model  ▲ | P_H_^*^ | Pooled RR  95%CI | P | Studies | N_E_/N_C_ | Model | P_H_ | Pooled RR  95%CI | P | Studies | N_E_/N_C_ | Model | P_H_ | Pooled RR  95%CI | P |
| TB | closure | 7 | 234/223 | F | 0.37 | 2.35(1.74,3.17) | <0.00001 | 5 | 177/175 | F | 0.67 | 1.59(1.34,1.88) | <0.00001 | 12 | 560/547 | R | 0.04 | 1.25(1.15,1.35) | <0.00001 |
|  | unchanged | - | - | - | - | - | - | - | - | - | - | - | - | 4 | 114/111 | F | 0.94 | 0.49(0.13,1.90) | 0.3 |
| TB+diabetes | closure | - | - | - | - | - | - | - | - | - | - | - | - | 2 | 65/52 | F | 0.39 | 1.38(1.09,1.74) | 0.007 |
| TB+HBsAg^+^ | closure | - | - | - | - | - | - | - | - | - | - | - | - | 2 | 77/58 | F | 0.84 | 1.17(1.02,1.34) | 0.02 |
| TB+pneumosilicosis | closure | - | - | - | - | - | - | - | - | - | - | - | - | 2 | 83/81 | F | 0.55 | 2.21(1.03,4.76) | 0.04 |
| Elderly TB | closure | 3 | 98/94 | F | 0.91 | 2.29(1.46,3.59) | 0.0003 | 2 | 31/18 | F | 0.47 | 1.61(1.14,2.27) | 0.007 | 4 | 111/105 | F | 0.64 | 1.2(0.98,1.46) | 0.07 |

#: N_E_ means the subject number of intervention group, N_C_ means the subject number of control group.

▲: F=Fixed model, R=Random model

*: P_H_ means the p value of heterogeneity test (α=0.05)
