# Supplementary material for: Mycobacterium vaccae as Adjuvant Therapy to Anti-Tuberculosis Chemotherapy in Never-Treated Tuberculosis Patients: A Meta-Analysis
Source: PLoS One. 2011 Sep 6;6(9):e23826. doi: 10.1371/journal.pone.0023826 (PMC3167806; doi:10.1371/journal.pone.0023826)
Supplement: Table S6 — Meta analysis of the levels of Th1/Th2 indicators. #: NE means the subject number of intervention group, NC means the subject number of control group. ▴: F = Fixed model, R = Random model. *: PH means the p value of heterogeneity test (α = 0.05). (DOC) [file pone.0023826.s006.doc]

Table S6 Meta analysis of the levels of Th1/Th2 indicators

|  | Indicators | Studies | N_E_/N_C_ ^#^ | Model  ▲ | P_H_^*^ | Pooled RR  95%CI | P |
| --- | --- | --- | --- | --- | --- | --- | --- |
| Th1 | IL-2 | 2 | 75/75 | F | 0.87 | 0.07(-0.25,0.39) | 0.65 |
|  | IFN-γ | - | - | - | - | - | - |
|  | TNF-α | 2 | 48/46 | R | <0.00001 | -11.03(-32.39,10.34) | 0.31 |
| Th2 | IL-4 | - | - | - | - | - | - |
|  | IL-6 | 2 | 75/75 | F | 1.00 | 0.01(-0.02,0.04) | 0.52 |

#: N_E_ means the subject number of intervention group, N_C_ means the subject number of control group.

▲: F=Fixed model, R=Random model

*: P_H_ means the p value of heterogeneity test (α=0.05)
